# Assessment of Eclipse electron Monte Carlo output prediction for various topologies

**DOI:** 10.1120/jacmp.v16i3.5036

**Published:** 2015-05-08

**Authors:** Shane L. Lawrence, Natascha H.M. Van Lieshout, Paule M. Charland

**Affiliations:** ^1^ Department of Physics University of Waterloo Waterloo ON Canada; ^2^ Medical Physics Grand River Hospital Kitchener ON Canada; ^3^ Electrical and Computer Engineering University of Waterloo Waterloo Ontario Canada

**Keywords:** electron Monte Carlo, MU calculation, topology, surface, output

## Abstract

Monte Carlo simulation is deemed to be the leading algorithm for accurate dose calculation with electron beams. Patient anatomy (contours and tissue densities) as well as irradiation geometry is accounted for. The accuracy of the Monitor Unit (MU) determination is one essential aspect of a treatment planning system. Patient‐specific quality assurance of a Monte Carlo plan usually involves verification of the MUs with an independent simpler calculation approach, in which flat geometry is to be assumed. The magnitude of the discrepancies between flat and varied surfaces for a few scenarios has been investigated in this study. The ability to predict MUs for various surface topologies by the commercial electron Monte Carlo implementation from Varian Eclipse system (Eclipse eMC) has been evaluated and compared to the Generalized Gaussian Pencil Beam (GGPB) algorithm. Ten phantoms with different topologies were constructed of water‐equivalent material. Measurements with a parallel plate ionization chamber were performed using these phantoms to gauge their relative impact on outputs for 6, 9, 12, 16, and 20 MeV electron beams from a Varian TrueBeam with cone sizes ranging from $6\times 6\;{\text{cm}}^{2}$ to $25\times 25\;{\text{cm}}^{2}$. The corresponding Monte Carlo simulations of the measured geometries were carried out using the CT scans of these phantoms. The results indicated that the Eclipse eMC algorithm can predict these output changes within 3% for most scenarios. However, at the lowest energy, the discrepancy was the greatest, up to 6%. In comparison, the Eclipse GGPB algorithm had much worse agreement, with discrepancies up to 17% at the lowest energies.

PACS numbers: 87.55.K‐, 87.55.km

## I. INTRODUCTION

Clinical electron beam dose calculations have undergone an evolution in recent years, becoming significantly more sophisticated.^(1)^ The article by Hogstrom and Almond^(1)^ reviews the evolution of algorithms for electron planning. Earlier methods for calculating dose from electron beams in clinical settings were based on measured data to which simple corrections for tissue heterogeneity and changes in the source‐to‐surface distance (SSD) could be introduced.^(2)^ Those treatment planning approaches were followed by pencil beam type algorithms whose main limitations are due to the central‐axis approximation. Recommendations for the characterization of clinical electron beams have been in place for many years.^3^, ^4^ A current trend exists to replace simpler algorithms with sophisticated Monte Carlo algorithms for computing electron dose distribution.^(5)^ The endeavor of the Monte Carlo algorithm is to model the complex transport of radiation across different media and geometry with substantial accuracy. There have been various evaluations of the Monte Carlo method for predicting dose^6^, ^7^, ^8^, ^9^, ^10^, ^11^ and in particular Zhang et al.^(11)^ presented a comprehensive evaluation of the Eclipse electron Monte Carlo algorithm, Eclipse eMC (Varian Medical Systems, Palo Alto, CA). It is a fast implementation or macro Monte Carlo algorithm.^(12)^ The algorithm uses precalculated EGS4^(13)^ data and generally provides good agreement with measurements.^6^, ^7^, ^8^, ^11^ Generally, a treatment planning algorithm evaluation starts with homogeneous water‐equivalent phantoms and standard geometries, after which more complex scenarios are added. It has been demonstrated that Eclipse eMC is able to predict small fields down to 3.0 cm diameter, over an energy range of 6 to 20 MeV, at a standard SSD of 100 cm within 2.5% accuracy.^(6)^ The Eclipse eMC was also shown to outperform the pencil beam algorithm with heterogeneous phantoms^(11)^ that incorporated lung‐ and bone‐equivalent material. The obliquity of incident beams is known to have an impact on the electron dose distribution.^14^, ^15^, ^16^ The obliquity or surface irregularity effect is also noted in the article from Zhang et al.^(11)^ In their article it is mentioned that the difference between the pencil beam and the Eclipse eMC algorithm predictions reached 7%.^(11)^ There is definitive evidence that the Eclipse eMC algorithm is sensing the contour changes, but it is not clear to what level of accuracy. There are some limited data with anthropomorphic phantoms on the impact of nonflat surface on predicted Eclipse eMC dose.^8^, ^11^ In the current article, output measurements in homogeneous, water‐equivalent phantoms of various topologies that could mimic different clinical situations have been performed. These measured output changes have been compared to the Eclipse eMC predictions. For comparison, the same scenarios are computed with the Eclipse Generalized Gaussian Pencil Beam (GGPB) algorithm.

## II. MATERIALS AND METHODS

### A. Phantom fabrication

**Figure 1:**
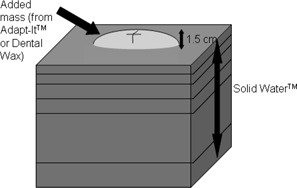
Phantoms with varied topology generated by adding a mass of water‐equivalent material at the surface of a Solid Water phantom. Added phantom masses had a height of 1.5 cm from the top of the Solid Water and were marked with a cross to indicate the isocenter.

**Figure 2:**
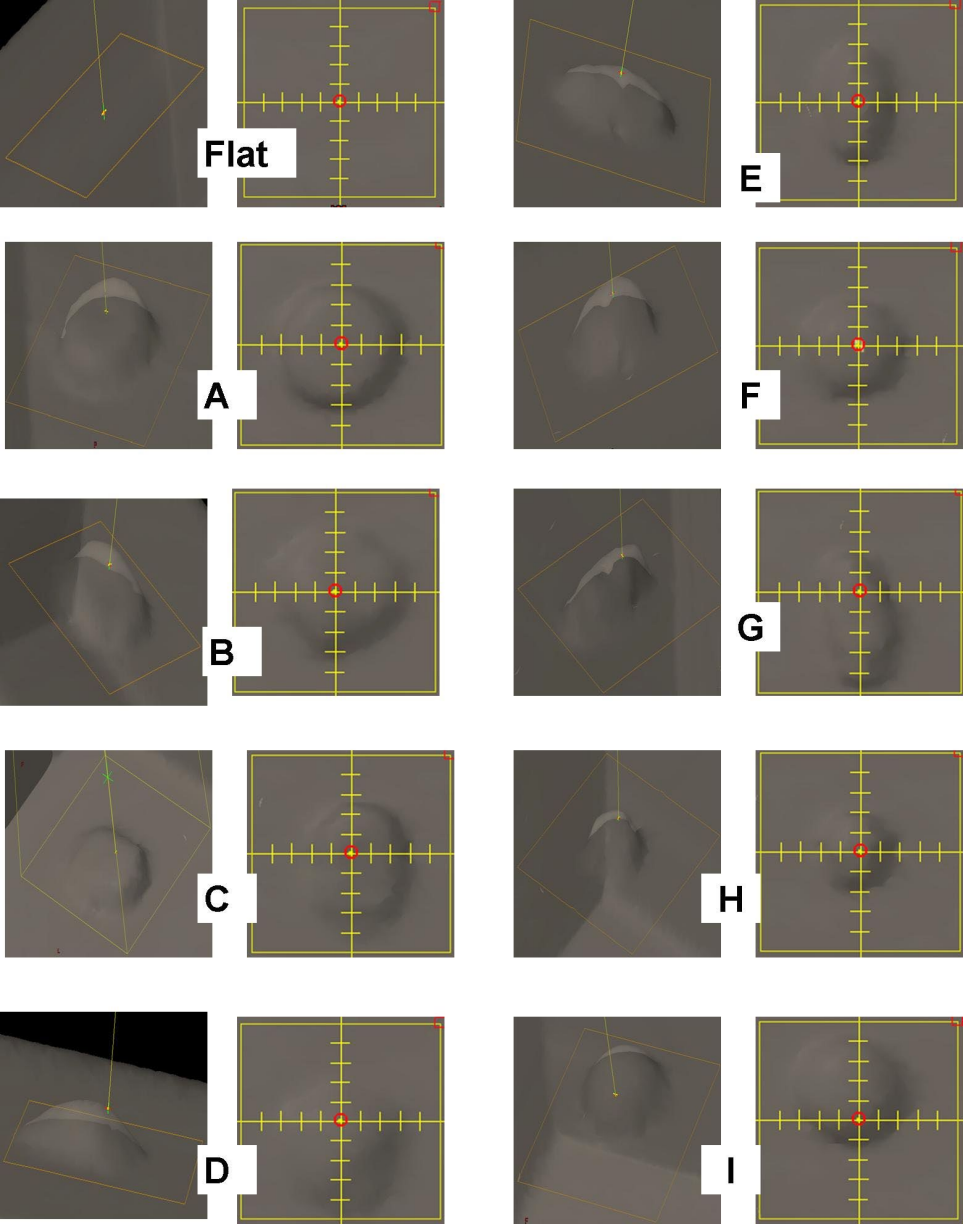
3D rendering of the ten phantoms as seen in the beam's eye view and with different perspective.

Ten phantoms with varied topologies were designed for validation purposes. The shapes and sizes somewhat mimic clinical presentation of breast scars and superficial growths. The first phantom had a flat geometry made from slabs of Solid Water (RMI, Gammex, Middleton, WI), a water‐equivalent material. The remaining phantoms were made of the same slabs to which varied rounded masses were added at the surface (Fig. 1). Seven of these masses were made of Adapt‐It thermoplastic material (Patterson Medical, Warrenville, IL), with a density of $1.1\;g/{\text{cm}}^{2}$, while the remaining two were made from dental baseplate wax (Radiation Products Design, Inc. Albertville, MN), with a density of $0.9\;g/{\text{cm}}^{2}$. Figure 2 shows a 3D rendering of the ten phantoms in the beam's eye view.

A cross was marked with a pen on each phantom to define where the central axis of the beams was to intercept. The location for the mark on the mass was selected such that the thickness of the added phantom mass was 1.5 cm at that point.

Radiopaque markers were positioned on the phantoms to indicate the isocenter for the CT simulations. All phantoms were scanned on a Siemens Sensation CT scanner, with 3 mm thick slices (Siemens Medical Solutions USA, Inc., Malvern, PA).

### B. Phantom irradiations

Irradiation of the ten different scenarios of phantom topology was performed on a Varian TrueBeam linac for 6, 9, 12, 16, and 20 MeV electron beams (Varian Medical Systems, Palo Alto, CA). These beams are matched to the factory reference dataset, the Varian Golden Beam data. The applicator factors were measured and are in agreement (within 0.8%) with data published in the literature for centers with similar linacs.^(17)^ The default jaw settings were used for the study.

The experimentation for this study consisted of point measurements inside these phantoms to assess their relative impact on the output as compared to flat geometry. For all the phantoms, the $10\times 10\;{\text{cm}}^{2}$ applicator was used and for a subset of phantoms, the remaining applicators, $6\times 6\;{\text{cm}}^{2}$, $15\times 15\;{\text{cm}}^{2}$, $20\times 20\;{\text{cm}}^{2}$, and $25\times 25\;{\text{cm}}^{2}$ were also tested. The SSD was always set to 100 cm. A parallel plate Markus ion chamber (PTW, Freiburg, Germany) inside a special Solid Water insert and a DOSE 1 electrometer (IBA Dosimetry, Bartlett, TN) provided the reading for the output measurements. The diameter of the window for the Markus chamber is 0.54 cm, its volume is 0.05 cm^3^, and the effective depth of the chamber is 1 mm from its surface. The effective depths of the measurements, which accounted for the Markus chamber thickness, were close to the nominal depths of maximum dose for the given energy (i.e., 1.6 cm for 6 MeV, 2.1 cm for both 9 MeV and 20 MeV, and 3.1 cm for both 12 MeV and 16 MeV). To achieve the measurement depths beyond 1.6 cm, Solid Water slabs were introduced as needed between the slab with the Markus chamber insert and the remaining phantom mass.

The relative output factor change, or correction factor $CF_{topo}$, due to topology effect for a given phantom with topology (topo) and an energy *E* has been defined as:

(1) $$CF_{topo}=ROF(topo,E)/ROF(flat,E)$$

where ROF(topo,E) and ROF(flat,E) are the relative output factors for a given phantom topology and a flat phantom, respectively, and a given electron energy *E*. With this definition, $CF_{topo}$ for a flat geometry is equal to unity.

For a subset of six phantoms, CBCT scans captured the image of the phantoms and the registered images were analyzed in Eclipse. Positioning error and reproducibility of the setup are estimated to be up to 2 mm.

### C. Eclipse calculations

The CT images of the phantoms were imported into the Eclipse treatment planning software system v10.0 (Varian Medical Systems). The beam modeling required minimum measured data and was set to be in close agreement with the Golden Data.^11^, ^17^ The BBs on the CT scan images were edited out of the body contour so as to be excluded from the dose calculation.

The grid size simulation parameters used for the Eclipse eMC calculations were the same as described by Popple et al.,^(8)^ — 1 mm for 6 and 9 MeV, 1.5 mm for 12 MeV, 2 mm for 16 MeV, and 2.5 mm for 20 MeV. An accuracy of 1% and low 3D Gaussian smoothing level were used for the Eclipse eMC calculations.^(6)^ To calculate the relative output factors (ROF) and correction factor (${\text{CF}}_{\text{topo}}$), the monitor units (MUs) for each plan were fixed to 100 MUs and dose to a point corresponding to the measurement geometry was read from the software. A comparison (results not shown) of volume averaging over the equivalent of the Markus chamber volume versus point‐dose extraction in Eclipse did not provide different information. The point‐dose comparison was chosen simply as it reflects current practice to independently verify MUs for the quality assurance of specific plans. The calculations with the GGPB algorithm v10.0 was also added to the comparison. The grid size was kept small for 6 and 9 MeV (0.125 cm), and 0.25 cm was used for the remaining electron energies.

In addition to the simulated phantoms, four clinical electron cases have been computed with Eclipse eMC. The same beams were recomputed on a flat phantom geometry. A ${\text{CF}}_{\text{topo}}$ factor was calculated for these clinical cases to illustrate the magnitude of this factor for clinical scenarios.

## III. RESULTS & DISCUSSION

### A. Comparison of Eclipse calculations and measurements

For a subset of six phantoms, ${\text{CF}}_{\text{topo}}$ measurements were obtained for all cone sizes. By definition, ${\text{CF}}_{\text{topo}}$ is normalized to the flat geometry of the same cone size. The variation of ${\text{CF}}_{\text{topo}}$ among the cone sizes for the various energies was found as expected to be negligible, within 0.3% difference. Eclipse eMC calculated CF topology for a given energy had a standard variation slightly higher, but within 1% between field sizes. These results confirm the suitability of Eq. (1) where the topology effects are decoupled from the field size effects. Hence for the remainder of the study, the impact of the different phantoms was studied for the $10\times 10\;{\text{cm}}^{2}$ cone only.

**Figure 3:**
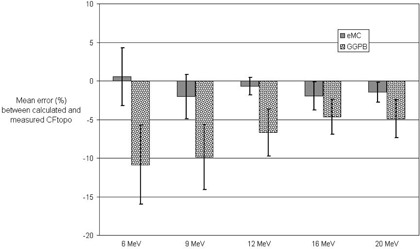
Mean errors in percentage (averaged over all ten phantoms) between the correction factors ${\text{CF}}_{\text{topo}}$ from Eclipse eMC calculations and the measurements. The mean errors are also presented for the GGPB algorithm for comparison. The standard deviations on the means are depicted with the error bars. Energies ranging from 6 MeV to 20 MeV, SSD 100 cm, and $10\times 10\;{\text{cm}}^{2}$ cone used.

Figure 3 presents the mean errors between the Eclipse eMC algorithm and the measurements of ${\text{CF}}_{\text{topo}}$, averaged over the ten phantoms. For comparison the mean errors between Eclipse GGPB and the measurements of ${\text{CF}}_{\text{topo}}$ are also shown. The error bars in Fig. 3 represent the standard deviations on the means. The mean error between Eclipse eMC and the measurement of CFtopo is generally small (i.e., within 2%) for all energies (Fig. 3). The lowest energy, 6 MeV, had the highest standard deviation of 3.7% for the mean error on ${\text{CF}}_{\text{topo}}$ with Eclipse eMC. The GGPB algorithm showed large mean errors between the calculated and measured ${\text{CF}}_{\text{topo}}$ and also had the highest standard deviation on the mean (5.1%) happening for 6 MeV.

**Figure 4:**
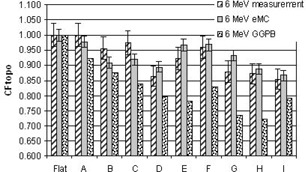
CF_topo_ factors as measured on the linac and predicted with the Eclipse eMC and the GGPB algorithms for 6 MeV electron beam and ten different phantoms.

4, 5, 6, 7, 8 illustrate the correction factors due to topology effects (${\text{CF}}_{\text{topo}}$) for various phantoms, the five electron energies, and $10\times 10\;{\text{cm}}^{2}$ applicator. The highest discrepancy between Eclipse eMC predicted and measured ${\text{CF}}_{\text{topo}}$ occurred at the lowest energy (6.1% discrepancy for 6 MeV, phantom G, Fig. 4). In contrast, the largest difference between calculated and measured ${\text{CF}}_{\text{topo}}$ reached −16.9% for the GGPB algorithm (Fig. 4, phantom H).

**Figure 5:**
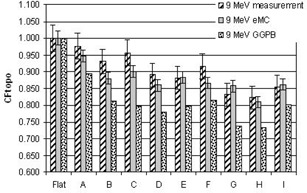
CF_topo_ factors as measured on the linac and predicted with the Eclipse eMC and the GGPB algorithms for 9 MeV electron beam and ten different phantoms.

**Figure 6:**
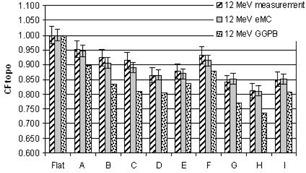
CF_topo_ factors as measured on the linac and predicted with the Eclipse eMC and the GGPB algorithms for 12 MeV electron beam and ten different phantoms.

**Figure 7:**
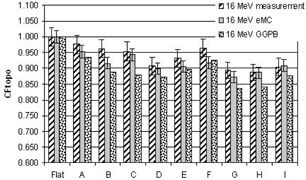
CF_topo_ factors as measured on the linac and predicted with the Eclipse eMC and the GGPB algorithms for 16 MeV electron beam and ten different phantoms.

**Figure 8:**
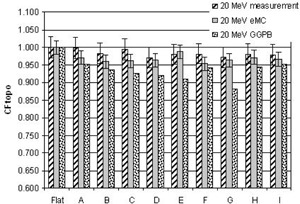
CF_topo_ factors as measured on the linac and predicted with the Eclipse eMC and the GGPB algorithms for 20 MeV electron beam and ten different phantoms.

For flat phantoms, ${\text{CF}}_{\text{topo}}$ is equal to unity by definition. For this class of phantom shapes that can mimic different clinical masses with smooth changes at the skin interface, it can be seen that the ${\text{CF}}_{\text{topo}}$ factor often dropped around 90%–95% due to contour change. The most dramatic changes in relative outputs due to varied topologies were observed at the lowest energies. For 6, 9, and 12 MeV (4, 5, 6) the measured correction factor ${\text{CF}}_{\text{topo}}$ dropped by close to 80% (CF_topo_ of 0.812 for 12 MeV). These output changes due to topology effects were corroborated by both measurements and Eclipse eMC calculations. The GGPB algorithm systematically underestimated ${\text{CF}}_{\text{topo}}$ for the nonflat phantoms (4, 5, 6, 7, 8).

The challenge to this study is the fact the electron dose gradients are inherently very sharp, especially for the low energies, and can be very noisy in Monte Carlo simulations. The uncertainties in this study are compounded by the fact that the phantoms themselves have smooth gradients which add depth uncertainty with any positioning error. Error bars of 4% for the 6 and 9 MeV measurements have been used in this study, and 3% for the highest energies based on positioning error estimation with CBCT and implications based on the dose gradient. As for the Eclipse eMC data, an error bar of 2% was set to reflect the combine uncertainties. The fit of the body contour to the phantom and some density effects could have some influence on the results.

### B. Clinical Cases

Table 1 contains the calculated values for ${\text{CF}}_{\text{topo}}$ based on the Eclipse eMC algorithm predicted doses for a few clinical scenarios. Results illustrate that it is not uncommon to encounter topological effects on the order of 6%–8% deviation from the flat scenario.

**Table 1:** ${\text{CF}}_{\text{topo}}$ for a few clinical situations based on the Eclipse eMC algorithm.

	*Case*	*Energy (MeV)*	$CF_{topo}\;eMC$
1	Scalp	12	0.924
2	Ear	12	0.935
3	Chest wall boost	9	0.932
4	Ear	16	0.936

This study was concerned only by the overall output change based on a point measurement, which has an impact on the overall scaling of the dose distribution and is a main parameter verified during routine patient‐specific quality assurance. It is understood that the surface topology impacts the entire dose distribution. The loss of side scatter equilibrium due to the irregular surface causes hot and cold spots in the dose distribution. Options for dealing with contours to try to achieve a straight incidence include careful selection of beam angle, splitting the beam into multiple fields up to electron arc, if it is available, or to have a custom bolus made. It is the experience of the authors nevertheless that there will remain many clinical scenarios with variable surfaces to be dealt with for which the accuracy of the dose will rely solely on the algorithm.

## IV. CONCLUSIONS

This is a study to describe the impact on output change for various anatomical shapes and electron energies, along with the ability of Eclipse eMC algorithm to predict it. It is demonstrated that the Eclipse eMC algorithm is able to reasonably predict the output dose for many varied surface topologies. These preliminary observations show that the Eclipse eMC algorithm can tackle the topology problem within 3% accuracy for most scenarios, but occasionally demonstrates greater uncertainty, up to 6%, for the lowest energies encountered. Uncertainties due to the dose gradients involved and the positioning uncertainties make this study challenging. Nevertheless, care was taken throughout this study to reflect the traditional practice of verifying MUs based on a representative point. Data showed the marked impact of topology change which, if unaccounted for, would lead to an unintended dosage. The weakness of the GGPB was also demonstrated in this study. This is a first set of phantoms with various topologies to test the electron algorithm. It is suggested that commissioning procedures should incorporate similar tests with datasets of increased level of sophistication to understand the full ability of the algorithm.

## Supporting information

Supplementary Material

Supplementary Material

Supplementary Material

Supplementary Material

Supplementary Material

Supplementary Material
