# Supplementary figures and images for: Assessment of Eclipse electron Monte Carlo output prediction for various topologies
Source: J Appl Clin Med Phys. 2015 May 8;16(3):99–106. doi: 10.1120/jacmp.v16i3.5036 (PMC5690142; doi:10.1120/jacmp.v16i3.5036)

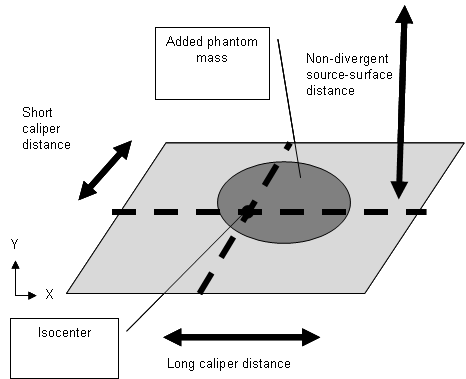

Supplement: Supplementary file 1 — Supplementary Material [file ACM2-16-099-s001.png]

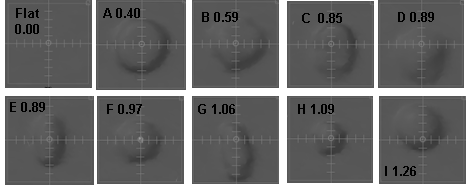

Supplement: Supplementary file 2 — Supplementary Material [file ACM2-16-099-s002.png]

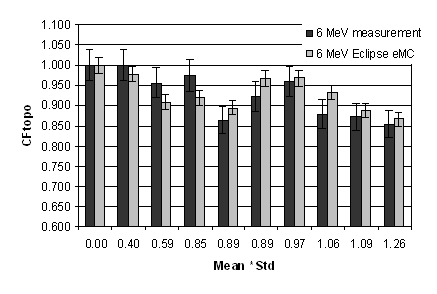

Supplement: Supplementary file 3 — Supplementary Material [file ACM2-16-099-s003.PNG]

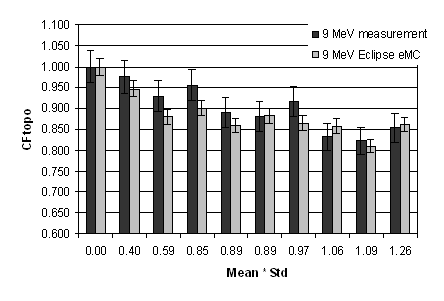

Supplement: Supplementary file 4 — Supplementary Material [file ACM2-16-099-s004.PNG]

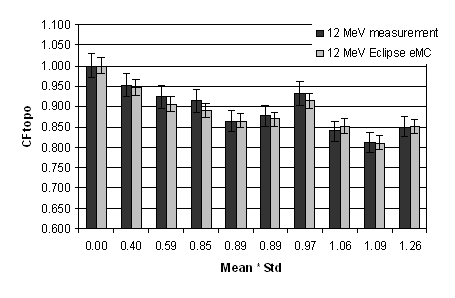

Supplement: Supplementary file 5 — Supplementary Material [file ACM2-16-099-s005.PNG]

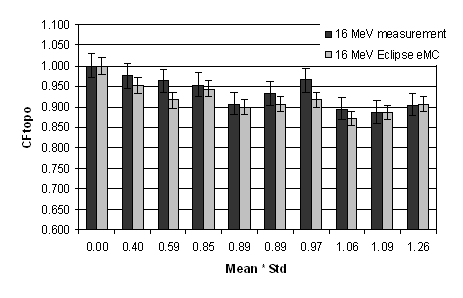

Supplement: Supplementary file 6 — Supplementary Material [file ACM2-16-099-s006.PNG]
